# An evolutionary game study on the collaborative governance of environmental pollution: from the perspective of regulatory capture

**DOI:** 10.3389/fpubh.2023.1320072

**Published:** 2024-01-05

**Authors:** Zikun Hu, Yina Wang, Hao Zhang, Wenjun Liao, Tingyu Tao

**Affiliations:** ^1^School of Economics and Management, Huaibei Normal University, Huaibei, China; ^2^Center for Industrial and Business Organization, Dongbei University of Finance and Economics, Dalian, China

**Keywords:** environment and public health, environmental pollution, regulatory capture, collaborative governance, four-party evolutionary game

## Abstract

Local governments have been captured by enterprises and, thus, have relaxed environmental regulations. This phenomenon has occurred repeatedly and has resulted in serious environmental pollution, posing an enormous threat to public health. To solve this problem, this study introduces central environmental protection inspection and media supervision and considers the economic preferences and environmental preferences of local governments. A four-party evolutionary game model composed of enterprises, local governments, the central government and the media is constructed, and the equilibrium solution of four-party replicator dynamics equations is obtained. The influence of relevant parameters on the choice of strategies of the four main bodies is simulated by using MATLAB software to explore the paths and measures for overcoming regulatory capture and to further improve the modern environmental governance system. The results show the following: First, local governments are easily captured by large enterprises. Second, the central government can improve the environmental behavior of local governments by reducing their economic preferences and strengthening punishment. Third, compared to the penalties imposed by the central government, those imposed by local governments have a more significant impact on the environmental behaviors of enterprises. Fourth, compared to the use of an environmental protection tax policy or a tax relief policy alone, the combination of the two has a more significant impact on the environmental behaviors of enterprises. Fifth, central environmental protection inspection and media supervision can improve the environmental behaviors of both local governments and enterprises, and the effect of media supervision is better than that of central environmental protection inspection. This study recommends improving the performance evaluation system for local governments to coordinate economic development and environmental protection, ensuring that local governments assume the main responsibility, using a combination of incentive and constraint policies for enterprises, and increasing the environmental protection inspection and media supervision of local governments and enterprises to resolve the dilemma of regulatory capture in environmental pollution through the simultaneous enhancement of the environmental behavior of local governments and enterprises.

## 1 Introduction

“Lucid waters and lush mountains are invaluable assets.” As China’s economy enters the stage of high-quality development, environmental pollution is increasingly receiving widespread attention from governments at all levels and the public (1). Effective regulation and control of environmental pollution are an important part of ensuring environmental protection and public health and safety (2, 3). In the report to the 20th National Congress of the Communist Party of China (CPC), General Secretary Xi Jinping proposed promoting green development, further promoting the prevention and control of environmental pollution, and promoting the harmonious coexistence of humans and nature, which raised ecological civilization construction to an unprecedented political height. As of January 1, 2018, the “Environmental Protection Tax Law of the People’s Republic of China” has been in effect, and an environmental tax, which is the first tax type levied in China for the purpose of environmental protection, was officially introduced (4, 5). In 2020, the “Guiding Opinions on Building a Modern Environmental Governance System” issued by the General Office of the CPC and the General Office of the State Council clearly noted that the key to environmental protection is strengthening the leading role of the government. The fundamental goal is to deepen the role of enterprises and to better mobilize both social organizations and the public to participate in supporting the implementation of the ecological and environmental protection inspection system at the central and the provincial level (the autonomous regions and municipalities directly under the Central Government) to deepen the inspection of ecological and environmental protection. This would strengthen the supervision of public opinion and encourage the news media to report on all kinds of ecological and environmental problems, environmental emergencies and environmental violations. The State Council also indicated that in addition to major environmental governance mistakes, such as national mistakes, key regional water basin mistakes, cross-regional mistakes, and mistakes involving international cooperation, local governments are mainly responsible for environmental governance expenditures (6).

These policies are characterized by the downward transfer of administrative and financial rights and serve to provide local governments with greater flexibility in environmental law enforcement (6, 7). At present, local officials are still faced with an official promotion and assessment system that is focused on economic development (8), and when carrying out environmental pollution remediation, local governments’ environmental regulatory objectives are often in conflict with GDP development indicators, such as local economic development, local employment levels, and fiscal revenues (9). Therefore, when local governments consider responses such as damaged economic development and a sharp drop in tax revenue that occur in the process of environmental law enforcement, they are forced to focus on economic development. It is possible to “turn a blind eye” or impose only minor penalties on the pollution violations of enterprises, which would result in the phenomenon of lax law enforcement or of law enforcement that only goes through the motions (10). For example, in 2018, a carbon 9 leak accident occurred in Quanzhou, Fujian, China. Initially, when residents in the Quangang District experienced physical discomfort and complained to the government, local government law enforcement departments not only did not impose severe punishment but also concealed the true situation of the incident. It was not until the media paid attention and the incident gradually developed and grew online that the local government intervened in the investigation and imposed severe punishment. In April 2022, the First Ecological and Environmental Protection Supervision Team of the Central Committee inspected Hebei Province and found that some enterprises in Xingtai, Tangshan and Zaoqiang had been disposing of sludge in violation of regulations for a long time, despite the fact that the cities and counties in Xingtai, Tangshan and Zaoqiang had strict regulations on sludge disposal. The lack of supervisory responsibility, inaction and slow action are the prominent problems that have enabled this illegal disposal of sludge for such a long time. It is very common for local governments to be captured by regulation, which is one of the more important reasons why China’s environmental governance is facing difficulty. Therefore, in the context of the reality of central environmental protection inspection and media supervision, clarifying the behavioral strategies of stakeholders such as enterprises, local governments, the central government and the media and exploring the causes of, mechanisms of, influencing factors of and effective preventive measures for regulatory capture hold important theoretical significance and practical value for resolving the dilemma of China’s environmental governance in the current stage.

## 2 Literature review

### 2.1 Environmental regulation

In recent years, the reformation of the vertical management of environmental protection has been continuously deepened, which is of great importance to studying the effect of environmental regulation policy on enterprises (11, 12). The innovation effect of environmental regulation has received widespread attention in academia. Some scholars are committed to exploring the impact that mandatory environmental laws and regulations have on technological improvement and green innovation (13, 14). Most people believe that well-designed mandatory environmental laws and regulations can promote enterprise environmental protection initiatives, thereby promoting the progress of production technology and green innovation (15–17). Strict and appropriate environmental regulation can not only reduce environmental pollution but can also indirectly promote coordinated economic development through mediating factors (18–21). An effective environmental policy tool balances the interests of the government, enterprises and the public, improves the environmental regulation standards, increases the penalties for illegal pollutant discharges by enterprises, exerts a positive influence on the strategic choices of local governments, enterprises and the public, and can leveraged to effectively solve the problems involved in the internalization of environmental externalities (22–24). Zhang and Zhao concluded that environmental regulation has a positive impact on low-carbon transition through the mechanisms of technological innovation, resource reallocation and skill premium (25). Some scholars believe that the reinforcing effect of environmental regulation on enterprise environmental governance behavior mainly impacts large enterprises and nonstate-owned enterprises (26). In addition, market-based environmental regulation exerts a great impact on the distribution pattern of factor income. Although environmental protection taxes can encourage enterprises to make green innovation and environmental protection investments, such taxes can also increase the uncertainty in enterprise innovation investment (27). Some scholars believe that from a static perspective, more stringent environmental regulations are not conducive to the improvement of production technologies and the transformation of production processes and can also lead enterprises to increase output and pollution emissions in pursuit of profit maximization (28, 29), thus hindering the high-quality development of industry and energy (30, 31).

### 2.2 Regulatory capture

“Regulatory capture theory” explains the original design intention and implementation effects of government regulation from the perspective of group sectoral interests, emphasizing that policy regulation is, in essence, a redistribution process of economic resources that proceeds through political decision-making and the “rent-seeking” behavior of special interest groups (32, 33). This theory originated from Stigler’s questioning of traditional regulatory theory in 1962 and forwards the claim that the original intention of regulatory policy was to meet the supervisory needs of regulated enterprises. Manish et al. confirmed the claim that the regulatory department will eventually be captured by the regulated (34). The regulatory resources enjoyed by the government also endow it with special powers. Regulators are motivated by self-interest (35), and they can rationally make choices to maximize their own utility.

Stigler groundbreakingly proposed that the government is prone to being bought by interest groups in the process of implementing regulatory policy. Through the capture of the government, interest groups create a vast amount of policy and institutional distortions to obtain excess profits for specific sectors or individuals, resulting in unsatisfactory regulatory effects and the emergence of regulation capture problems (36). On the basis of Stigler’s work, Petzman further constructed a theoretical model to describe regulatory capture. Under the assumption that interest groups and the regulatory department both comprise economic agents who pursue the maximization of their own interests, the regulatory policy supply can be seen to be the relationship between these interest groups and the institutional arrangements that regulate the common preferences of these departments (37). Ponser proposed that even if the original intention of regulatory policy is to ensure the public interest, the results of regulatory implementation often deviate from this original intentions due to the influence of interest groups (38). Laffont and Tirol brought the issue of information asymmetry into the analytical framework of regulatory capture and used the principal-agent structure under the condition of information asymmetry to investigate the formation mechanisms and factors influencing regulatory capture (39). In recent years, scholars have begun to give attention to the phenomenon of regulatory capture in China. The study by Lei et al. showed that the actual control of resources is prone to result in regulatory capture between environmental protection inspection departments and enterprises (40). In terms of the causes of regulatory capture, Yuan concluded that policy burden is the main factor leading to regulatory capture. When the policy burden exceeds a certain threshold, it results in regulatory capture and hinders green innovation (10). In terms of the consequences of regulatory capture, Xu et al. concluded that regulatory capture negatively regulates the promoting effect of environmental regulation on R&D and innovation (41). Feng et al. analyzed the effect of regulation capture on environmental efficiency in the institutional environment. Based on this mechanism, they concluded that regulatory capture significantly inhibits improvements in regional environmental efficiency (42). In terms of prevention strategies for regulatory capture, Yuan et al. considered both local government preferences and the environmental tax system and found that environmental inspection by the central government is the key to avoiding regulatory capture (43). Zhao introduced regulatory capture theory and media governance theory into the analysis of environmental regulatory issues, constructed a game model composed of polluting enterprises and local environmental regulators for theoretical analysis, and concluded that media exposure of environmental pollution incidents in the jurisdiction of the polluting enterprise often attracts the attention of public opinion and the direct intervention of the relevant departments of the central government. Additionally, these concerns form enormous law enforcement pressure, which reduces the administrative burden of local environmental law enforcement departments and increases the probability that the government will directly intervene and severely crack down on violations (44). Some scholars have further proposed that in the process of “decentralization, regulation and services” reform, the government, by promoting internet supervision, developing credit supervision, and reducing approval items, can exert a positive preventive effect on the existing forms of environmental regulatory capture (45, 46).

### 2.3 Central environmental inspection

Central environmental inspection is an important supervisory system in the environmental field, and its direct target is the environmental governance behavior of local governments (47, 48). With the deepening of efforts to construct an ecological civilization, central government direct inspection has become the new normal for environmental protection inspection (49–51). Beginning in 2016, the central government implemented central environmental inspection across the country. The implementation of this policy has captured the interest of relevant scholars (52, 53). Wu and Hu, Wang et al. and Jia and Chen et al. used the difference-in-differences and discontinuity regression methods to study the practical effect of central environmental inspection, and their results generally revealed that central environmental inspection can improve environmental quality (54–56). However, the sustainability of this effect remains open to question. Many scholars have studied the mechanism by which central environmental inspection affects air pollution control. Their results show that while current environmental inspection can effectively motivate polluting enterprises to actively pursue rectifications, the incentive effect on local governments is limited (57, 58). The improvement in air quality in nonenvironmental protection-focused cities, however, outperforms that of key environmental protection cities (59, 60). In addition, the effectiveness of central environmental inspection is not sustainable (61, 62). Some scholars have studied the impact of central environmental inspection on enterprise green technology innovation and generally found that central environmental inspection significantly promotes the green technology innovation of pollution-intensive industrial enterprises (63, 64). At the micro level of enterprises, Qian et al. and Feng et al. evaluated the microgovernance effect of central environmental inspection and found that it increases the enterprise’s environmental protection expenditure and enhances the enterprise’s sense of social responsibility and environmental performance (65, 66).

### 2.4 The role of the media

China’s environmental governance results from the participation of multiple parties. In addition to traditional players such as the central government, local governments, enterprises and the public, the media, as an important external force, is gradually deepening and expanding its influence on local environmental governance. As of June 2022, China’s internet penetration rate reached 74.4% (67). The new media represented by the internet and the mobile internet are characterized by immediacy, openness, interactivity and public visibility (68). These media provide information support for public participation, create public space, and greatly enhance citizens’ right to speak and enthusiasm for participation (69). In particular, they have obvious advantages in promoting policy agendas, reuniting stakeholder groups, and pursuing innovation in the interaction and communication mechanisms between the government and the public (70, 71). This represents a novel way for the public to participate in environmental governance and plays a key role in communicating public expectations for environmental governance in the digital context (72, 73).

As an important topic of environmental governance, the media has the right to choose whether to conduct environmental supervision, and this choice often depends on the level of environmental supervision of local governments and the media’s own cost–benefit analysis (74). It is precisely in balancing these dynamics that the media exerts influence on local environmental governance. First, the media has wide coverage, strong penetration and great influence, which affects their level of investment in environmental governance (75, 76). Second, the media helps to construct public opinion on the national ecological environment, to construct a discourse system, and to induce and guide the audience’s environmental cognition. This also reflects the subjectivity of the media in the construction of public opinion (77). Finally, the media play a role in the supervision of enterprises. Media reports amplify corporate social responsibility and promote prudent production, thereby actively promoting environmental research and development (78).

Li et al. concluded through their empirical research that media attention has increased the total investment level of local governments in environmental pollution control (79). Chang et al. concluded that media attention can promote enterprises’ environmental protection investment (80). Wang and Zhang fully affirmed the positive role played by media supervision and pressure in environmental protection and governance (81). The studies of Dasgupta et al., which are based on samples of developing countries such as Argentina, Chile, and Mexico, showed that the disclosure of an enterprise’s environmental pollution incident by the media exerts an impact on that enterprise’s performance in the capital market, thereby inhibiting enterprises’ environmental behavior (82). Gupta and Goldar reached similar conclusions based on research in India (83).

Therefore, through the study of the media influence on local governments’ environmental governance, expanding the decision-making mechanism underlying social governance can provide a theoretical basis and practical guidance for promoting the coordinated development of the economy and the environment.

### 2.5 Study on the applicability of game theory

As an important theoretical tool of information economics, evolutionary game theory breaks through the assumption of complete rationality in traditional game theory, and can be more reasonably used to describe various game interactions in the real world on the premise of bounded rationality. In the process of strategy selection, the result of equilibrium is to reach the equilibrium state through continuous trial and error, adjustment and improvement. Based on the advantages of bounded rationality and evolutionarily stable strategy, many scholars have used evolutionary game theory to conduct valuable research on multiagent games and their strategy selection behavior in environmental regulation. For example, Zhu et al. considered the rent-seeking phenomenon of enterprises in pollution prevention and control, constructed a tripartite evolutionary game model among enterprises, local governments and the central government, and analyzed the evolutionary stability of the strategic choices of each participant (84). Fan et al. established an evolutionary game model to analyze the operating mechanism of the different expenditure preferences of local governments on the production behavior of industrial polluting enterprises, and they conclude that whether the relationship between local governments and polluters in environmental governance is cooperative or collusive depends on the order of their games and initial endowments (85). Wang et al. established one tripartite evolutionary game model to investigate the decision-making mechanism of enterprises, the public and the government in the process of air pollution control in China. The results show that collaborative governance among these three stakeholders forms the optimal path for air pollution control in China (86). Xu et al. established a tripartite evolutionary game model consisting of the government, environmental service companies and pollutant discharging enterprises and reached the conclusion that the “public–private partnership” model is the key to increasing environmental public interests in the process of realizing economic interests (87). Wei et al. constructed a tripartite evolutionary game model that uses pollution control companies, professional environmental inspection agencies and government regulatory departments as the main bodies. The results of the study show that the government can effectively regulate the behavior of pollution control companies and professional environmental inspection agencies by appropriately increasing the relevant rewards and punishments, but excessive rewards are unfavorable for improving the performance of government regulatory agencies themselves (88).

In recent years, in the field of environmental governance, a small number of researchers have gradually attempted to use the four-party evolutionary game method to conduct studies on environmental issues, which has further expanded the application research of the game method. Pan et al. constructed a four-party evolutionary game model of local environmental governance by using the central government, local government, pollutant discharging enterprises and the public as the players. Their study finds that media attention strengthens the status of local governments and enterprises as key players and mainly affects the stability of the equilibrium point by affecting the environmental behavior of the two parties while having no effect on the strategic choice of the central government (67). Liu and Li constructed an evolutionary game model consisting of small and medium-sized livestock and poultry farmers, third-party companies, consumers, and local governments and concluded that the main factors affecting third-party recycling treatment include government regulation, the market demand for environmental conservation services and the market demand for organic fertilizers (89).

The method of evolutionary game has developed rapidly in recent years and has unique advantages in dealing with environmental problems. Therefore, the method of evolutionary game provides sufficient ideas for the problems to be solved in this study, and this study will make full use of the method of evolutionary game to model and analyze the problems to be solved.

The literature above shows that existing research has the following shortcomings: (1) In terms of the regulation of local governments and the regulation of enterprises, current scholars mainly focus on studying a single approach, such as media supervision or central environmental protection inspection (42, 43), and few scholars include media supervision and central environmental protection inspection into the same model for study. In terms of the environmental behavior of enterprises, few scholars quantitatively study the effect of the combination of incentive-based and constraint policies. In addition, the environmental behavior of local governments mainly involves economic preferences (8), reward and punishment mechanisms (88), policy burdens (10), and environmental protection tax policies (6, 42), and few scholars have considered both the economic and environmental preferences of local governments. (2) In terms of the relevant subjects involved in environmental pollution governance, existing studies mainly include the central government (67), local governments (84), polluting enterprises (85), third-party environmental protection organizations (87), the public (86), and the media (79), and few scholars include enterprises, local governments, the central government, and the media in the same model to study environmental pollution governance.

In summary, the innovations of this study are as follows: The first innovation involves the research perspective. Media supervision and central environmental protection inspection are included in the same model to study the effect of the two on overcoming environmental regulatory capture. Incentive-based policies and constraint policies are included in the same model for policy combination research. In addition, with environmental performance being included in the assessment, local government decision-making is affected by environmental utility. Therefore, this study also considers both the economic and environmental preferences of local governments, effectively portraying the utility function of local government behavior and making it closer to reality. The second innovation involves the research framework. In the study of environmental regulatory capture, enterprises, local governments, the central government and the media are included in the same research framework. A game model consisting of the four parties above is established, and regulatory capture is combined with the four-party evolutionary game. A systematic analysis of China’s environmental governance dilemma in regard to the formation of the mechanism, the influencing factors and the effective preventive measures is conducted. Through numerical simulation, the strategy optimization path of four-party synergistic management of environmental pollution is presented to provide a theoretical reference and practical guidance for the government’s policymaking.

## 3 Model construction and analysis

### 3.1 Background description

In the process of environmental pollution control, the enterprise is deemed the main source of pollution, and the ultimate goal is to maximize enterprise interests. At the same time, the enterprise is subject to two levels of supervision, one from the central government and one from local governments. The central government formulates corresponding environmental policies to guide specific environmental governance behaviors, and its main objectives are to safeguard social public interests and maximize overall societal welfare. The environmental policies formulated by the central government are implemented and executed by local governments. Local governments are rational economic persons with regional interests. First, local governments must follow the will of the central government and control local environmental pollution in accordance with the policies issued by the central government. On the other hand, in the case of promotion and economic performance assessment, local governments may go against the will of the central government and be captured by enterprises. This, in turn, cause them to acquiesce to the pollution discharge behavior of enterprises or mitigate the penalties levied for such behavior. In the current context of the rapid development of the internet, new media is playing an increasingly important role in the supervision of environmental governance. Many pollution incidents are first exposed by the media prior to being handled by the government, which eventually promotes the control of environmental pollution.

### 3.2 Model assumptions

*Hypothesis 1*: The game players in this study are enterprises, local governments, the central government and the media. Due to unavoidable factors such as information insufficiency, information asymmetry, and individual knowledge boundaries, the four game agents are all considered bounded rational agents, the four game agents achieve dynamic equilibrium through the processes of continuous learning, adaptation, and imitation, and the strategy selection is gradually optimized over time.

*Hypothesis 2*: The strategies that an enterprise can adopt include “pollution control” and “no pollution control.” When the enterprise does not control its pollution but rather maintains its original production, its income is $R_{1}$ , and the sewage discharge volume is $Q_{1}$ . The income of the enterprise after pollution control and emission reduction is $R_{2}$ , and the sewage discharge volume is $Q_{2}$ . The enterprise’s pollution control and emission reduction cost is $C_{1}$ , and in the actual situation, $R_{1}>R_{2}$ and $Q_{1}>Q_{2}$ .

*Hypothesis 3*: The strategies that local governments can adopt include “active implementation” and “passive implementation.” The cost of local governments’ active implementation of environmental policy is $C_{2}$ , the environmental protection tax rate is α , the tax reduction ratio of local governments for pollution control and emission reduction in enterprises is β , and the enterprise that does not control its pollution is fined in the amount of $F_{1}$ . When the local government negatively implements environmental policies, the implementation cost is $aC_{2}$ , and both the tax reduction ratio and the penalty amount for the enterprise, which are aβ and $aF_{1}$ , respectively, are reduced, where $a\left(0<a<1\right)$ indicates the implementation level of the local government’s environmental policy. The gains of local governments are mainly economic gains and environmental gains. The economic gains of local governments are $p_{1}R_{1}$ or $p_{1}R_{2}$ , where $p_{1}$ represents the economic preference of the local government. When the local government actively implements environmental policy, it expects negative benefits, such as the hindrance of local economic development and a large increase in the number of unemployed individuals in the short term. At this time, the economic benefit of the local government drops to $p_{1}R_{1}^{\prime }$ . The environmental benefits of the local government are $p_{2}Q_{1}$ or $p_{2}Q_{2}$ , and $p_{2}$ represents the environmental preference degree of the local government. The degree of the economic preference and environmental preference of local governments is mainly determined by the local government performance appraisal system established by the central government, but it is also influenced by the subjective preferences of local officials.

*Hypothesis 4*: The strategies that the central government can adopt include “inspection” and “no inspection.” The inspection cost to the central government is $C_{3}$ , and the utility of the central government mainly comes from two aspects: economic development and environmental protection. The economic development coefficient and environmental protection coefficient are $v_{1}$ and $v_{2}$ , respectively. To avoid regulatory capture, when the enterprise fails to control its pollution but the local government actively implements the policy, the fine imposed by the central government on the enterprise is $F_{2}$ . When the local government passively implements the pollution control and emission reduction in enterprises, the political penalty of the central government on the local government is $F_{3}$ . When both the enterprise and the local government have violations, that is, regulatory capture occurs, the penalties from the central government to both parties increase by k times.

*Hypothesis 5*: The strategies the media can adopt include “exposure” and “nonexposure.” The media exposure cost is $C_{4}$ , and the probability that the media successfully discovers regulatory capture is μ . When the media exposes the violations of the enterprise and the local government, it exerts a negative impact on the reputations of both parties, e.g., the enterprise loses the priority right to enter the market, while the support of the public and the social credibility of the local government will both decline, and these are divided and denoted as $\theta B_{1}$ and $\theta B_{2}$ , where θ is the influence of media exposure. In addition, media supervision is funded by the general public, and this is set to G . Further, when the media successfully exposes regulatory capture, they are rewarded by the central government *H*.

*Hypothesis 6*: In the game played among the enterprise, the local government, the central government and the media, the probability of the enterprise adopting the pollution control strategy is x , and the probability of adopting the no pollution control strategy is 1−x . The probability of the local government adopting the “active implementation” strategy is y , and the probability of its adopting the “passive implementation” strategy is 1−y . The probability of the central government adopting the “inspection” strategy is z , and the probability of its adopting the “no inspection” strategy is 1−z . The probability of the media adopting the “exposure” strategy is m , and the probability of its adopting the “nonexposure” strategy is 1−m . Among them 0<x<1 , 0<y<1 , 0<z<1 and 0<m<1 . Table 1 lists the values and meanings of the parameters.

**Table 1:** Parameter definitions.

Parameters	Definition
$R_{1}$	Income earned by the enterprise without pollution control while maintaining its original production
$R_{2}$	Income from enterprises’ pollution control and emission reduction
$Q_{1}$	Volume of pollution discharged by the enterprise when it does not control its pollution
$Q_{2}$	Amount of pollutants discharged by the enterprise during pollution control
$C_{1}$	Enterprise pollution control and emission reduction costs
$C_{2}$	Costs of local governments’ active implementation of environmental policies
$C_{3}$	Inspection costs to the central government
$C_{4}$	Media exposure cost
$F_{1}$	Local governments’ fines on enterprises that do not control their pollution
$F_{2}$	Fines imposed by the central government on enterprises
$F_{3}$	Political punishment of local governments by the central government
α	Environmental tax rate
β	Proportion of local government tax reductions and exemptions for pollution control enterprises
a	Implementation extent of environmental policies by local governments
$p_{1}$	Economic preference of local governments
$p_{2}$	Environmental preference of local governments
$v_{1}$	Economic development coefficient of the central government
$v_{2}$	Environmental protection coefficient of the central government
μ	The probability of media successfully discovering regulatory capture
$B_{1}$	Reputational loss of enterprises
$B_{2}$	Reputational loss of local governments
θ	The influence of media exposure
G	Public funding for the media
H	Rewards offered by the central government to the media

Based on the assumptions above, the gains to the enterprise, local government, central government, and media under different behavioral strategies are shown in Table 2.

**Table 2:** Behavior and strategy combinations and benefits for enterprises, local government, central government and media.

Strategy combination	Enterprise	Local government	Central government	Media
(Pollution control, active implementation, inspection, and exposure)	$R_{2}-\left(1-\beta \right)\alpha Q_{2}-C_{1}$	$p_{1}R_{2}-p_{2}Q_{2}-C_{2}$	$v_{1}R_{2}-v_{2}Q_{2}-C_{3}$	$G-C_{4}$
(Pollution control, active implementation, inspection, and nonexposure)	$R_{2}-\left(1-\beta \right)\alpha Q_{2}-C_{1}$	$p_{1}R_{2}-p_{2}Q_{2}-C_{2}$	$v_{1}R_{2}-v_{2}Q_{2}-C_{3}$	0
(Pollution control, active implementation, no inspection, and exposure)	$R_{2}-\left(1-\beta \right)\alpha Q_{2}-C_{1}$	$p_{1}R_{2}-p_{2}Q_{2}-C_{2}$	$v_{1}R_{2}-v_{2}Q_{2}$	$G-C_{4}$
(Pollution control, active implementation, no inspection, and nonexposure)	$R_{2}-\left(1-\beta \right)\alpha Q_{2}-C_{1}$	$p_{1}R_{2}-p_{2}Q_{2}-C_{2}$	$v_{1}R_{2}-v_{2}Q_{2}$	0
(Pollution control, passive implementation, inspection, and exposure)	$R_{2}-\left(1-a\beta \right)\alpha Q_{2}-C_{1}$	$p_{1}R_{2}-p_{2}Q_{2}-aC_{2}-\mu \theta B_{2}-F_{3}$	$v_{1}R_{2}-v_{2}Q_{2}-C_{3}$	$G-C_{4}+H$
(Pollution control, passive implementation, inspection, and nonexposure)	$R_{2}-\left(1-a\beta \right)\alpha Q_{2}-C_{1}$	$p_{1}R_{2}-p_{2}Q_{2}-aC_{2}-F_{3}$	$v_{1}R_{2}-v_{2}Q_{2}-C_{3}$	0
(Pollution control, passive implementation, no inspection, and exposure)	$R_{2}-\left(1-a\beta \right)\alpha Q_{2}-C_{1}$	$p_{1}R_{2}-p_{2}Q_{2}-aC_{2}-\mu \theta B_{2}$	$v_{1}R_{2}-v_{2}Q_{2}$	$G-C_{4}$
(Pollution control, passive implementation, no inspection, and nonexposure)	$R_{2}-\left(1-a\beta \right)\alpha Q_{2}-C_{1}$	$p_{1}R_{2}-p_{2}Q_{2}-aC_{2}$	$v_{1}R_{2}-v_{2}Q_{2}$	0
(No pollution control, active implementation, inspection, and exposure)	$R_{1}-\alpha Q_{1}-F_{1}-F_{2}-\mu \theta B_{1}$	$p_{1}R_{1}^{\prime }-p_{2}Q_{1}+F_{1}-C_{2}$	$v_{1}R_{1}-v_{2}Q_{1}-C_{3}+F_{2}$	$G-C_{4}+H$
(No pollution control, active implementation, inspection, and nonexposure)	$R_{1}-\alpha Q_{1}-F_{1}-F_{2}$	$p_{1}R_{1}^{\prime }-p_{2}Q_{1}+F_{1}-C_{2}$	$v_{1}R_{1}-v_{2}Q_{1}-C_{3}+F_{2}$	0
(No pollution control, active implementation, no inspection, and exposure)	$R_{1}-\alpha Q_{1}-F_{1}-\mu \theta B_{1}$	$p_{1}R_{1}^{\prime }-p_{2}Q_{1}+F_{1}-C_{2}$	$v_{1}R_{1}-v_{2}Q_{1}$	$G-C_{4}$
(No pollution control, active implementation, no inspection, and nonexposure)	$R_{1}-\alpha Q_{1}-F_{1}$	$p_{1}R_{1}^{\prime }-p_{2}Q_{1}+F_{1}-C_{2}$	$v_{1}R_{1}-v_{2}Q_{1}$	0
(No pollution control, passive implementation, inspection, and exposure)	$R_{1}-\alpha Q_{1}-aF_{1}-kF_{2}-\mu \theta B_{1}$	$p_{1}R_{1}-p_{2}Q_{1}+aF_{1}-aC_{2}-kF_{3}-\mu \theta B_{2}$	$v_{1}R_{1}-v_{2}Q_{1}-C_{3}+kF_{2}$	$G-C_{4}+H$
(No pollution control, passive implementation, inspection, and nonexposure)	$R_{1}-\alpha Q_{1}-aF_{1}-kF_{2}$	$p_{1}R_{1}-p_{2}Q_{1}+aF_{1}-aC_{2}-kF_{3}$	$v_{1}R_{1}-v_{2}Q_{1}-C_{3}+kF_{2}$	0
(No pollution control, passive implementation, no inspection, and exposure)	$R_{1}-\alpha Q_{1}-aF_{1}-\mu \theta B_{1}$	$p_{1}R_{1}-p_{2}Q_{1}+aF_{1}-aC_{2}-\mu \theta B_{2}$	$v_{1}R_{1}-v_{2}Q_{1}$	$G-C_{4}$
(No pollution control, passive implementation, no inspection, and nonexposure)	$R_{1}-\alpha Q_{1}-aF_{1}$	$p_{1}R_{1}-p_{2}Q_{1}+aF_{1}-aC_{2}$	$v_{1}R_{1}-v_{2}Q_{1}$	0

### 3.3 Model construction

The enterprise’s expected gain when it chooses the ‘pollution control’ strategy is shown in equation (1):


(1) $$\begin{array}{c} U_{11}=yzm\left[R_{2}-\left(1-\beta \right)\alpha Q_{2}-C_{1}\right]+yz\left(1-m\right)\\ \left[R_{2}-\left(1-\beta \right)\alpha Q_{2}-C_{1}\right]+y\left(1-z\right)\\ m\left[R_{2}-\left(1-\beta \right)\alpha Q_{2}-C_{1}\right]+y\left(1-z\right)\left(1-m\right)\\ \left[R_{2}-\left(1-\beta \right)\alpha Q_{2}-C_{1}\right]+\left(1-y\right)\\ \mathrm{zm}\left[R_{2}-\left(1-\beta \right)\alpha Q_{2}-C_{1}\right]\\ +\left(1-y\right)z\left(1-m\right)\left[R_{2}-\left(1-\beta \right)\alpha Q_{2}-C_{1}\right]\\ +\left(1-y\right)\left(1-z\right)m\left[R_{2}-\left(1-\beta \right)\alpha Q_{2}-C_{1}\right]\\ +\left(1-y\right)\left(1-z\right)\left(1-m\right)\left[R_{2}-\left(1-\beta \right)\alpha Q_{2}-C_{1}\right] \end{array}$$

The enterprise’s expected gain when it chooses the ‘no pollution control’ strategy is shown in equation (2):


(2) $$\begin{array}{c} U_{12}=yzm\left(R_{1}-\alpha Q_{1}-F_{1}-F_{2}-\mu \theta B_{1}\right)+yz\left(1-m\right)\\ \left(R_{1}-\alpha Q_{1}-F_{1}-F_{2}\right)+y\left(1-z\right)m\left(R_{1}-\alpha Q_{1}-F_{1}-\mu \theta B_{1}\right)\\ +y\left(1-z\right)\left(1-m\right)\left(R_{1}-\alpha Q_{1}-F_{1}\right)+\left(1-y\right)\\ \mathrm{zm}\left(R_{1}-\alpha Q_{1}-aF_{1}-kF_{2}-\mu \theta B_{1}\right)+\left(1-y\right)z\left(1-m\right)\\ \left(R_{1}-\alpha Q_{1}-aF_{1}-kF_{2}\right)+\left(1-y\right)\left(1-z\right)\\ m\left(R_{1}-\alpha Q_{1}-aF_{1}-\mu \theta B_{1}\right)+\left(1-y\right)\left(1-z\right)\left(1-m\right)\\ \left(R_{1}-\alpha Q_{1}-aF_{1}\right) \end{array}$$

The enterprise’s average expected gain is shown in equation (3):


(3) $$\bar{U_{1}}=xU_{11}+\left(1-x\right)U_{12}$$

The replicator dynamics equation of the constructed enterprise is:


(4) $$\begin{array}{l} F\left(x\right)=dx/dt=x\left(1-x\right)(R_{2}-\alpha Q_{2}+\alpha \beta Q_{2}\\ \;-C_{1}+yzF_{2}\;+yF_{1}+zkF_{2}-yzkF_{2}\\ \;+m\mu \theta B_{1}-R_{1}+\alpha Q_{1}+aF_{1}-yaF_{1}) \end{array}$$

The local government’s expected gain when it chooses the ‘active implementation’ strategy is shown in equation (5):


(5) $$\begin{array}{c} U_{21}=xzm\left(p_{1}R_{2}-p_{2}Q_{2}-C_{2}\right)+xz\left(1-m\right)\\ \left(p_{1}R_{2}-p_{2}Q_{2}-C_{2}\right)+x\left(1-z\right)m\left(p_{1}R_{2}-p_{2}Q_{2}-C_{2}\right)\\ +x\left(1-z\right)\left(1-m\right)\left(p_{1}R_{2}-p_{2}Q_{2}-C_{2}\right)+\left(1-x\right)\\ \mathrm{zm}\left(p_{1}R_{1}^{\prime }-p_{2}Q_{1}+F_{1}-C_{2}\right)+\left(1-x\right)z\left(1-m\right)(p_{1}R_{1}^{\prime }-\\ p_{2}Q_{1}+F_{1}-C_{2})+\left(1-x\right)\left(1-z\right)m\left(p_{1}R_{1}^{\prime }-p_{2}Q_{1}+F_{1}-C_{2}\right)\\ +\left(1-x\right)\left(1-z\right)\left(1-m\right)\left(p_{1}R_{1}^{\prime }-p_{2}Q_{1}+F_{1}-C_{2}\right) \end{array}$$

The local government’s expected gain when it chooses the ‘passive implementation’ strategy is shown in equation (6):


(6) $$\begin{array}{c} U_{22}=xzm\left(p_{1}R_{2}-p_{2}Q_{2}-aC_{2}-\mu \theta B_{2}-F_{3}\right)\\ +xz\left(1-m\right)\left(p_{1}R_{2}-p_{2}Q_{2}-aC_{2}-F_{3}\right)+x\left(1-z\right)\\ m\left(p_{1}R_{2}-p_{2}R_{2}-aC_{2}-\mu \theta B_{2}\right)+x\left(1-z\right)\left(1-m\right)\\ \left(p_{1}R_{2}-p_{2}Q_{2}-aC_{2}\right)+\left(1-x\right)\mathrm{zm}(p_{1}R_{1}-p_{2}Q_{2}\\ +aF_{1}-aC_{2}-kF_{3}-\mu \theta B_{2})+\left(1-x\right)z\left(1-m\right)\\ \left(p_{1}R_{1}-p_{2}Q_{1}+aF_{1}-aC_{2}-kF_{3}\right)+\left(1-x\right)\left(1-z\right)\\ m\left(p_{1}R_{1}-p_{2}Q_{1}+aF_{1}-aC_{2}-\mu \theta B_{2}\right)+\left(1-x\right)\\ \left(1-z\right)\left(1-m\right)\left(p_{1}R_{1}-p_{2}Q_{1}+aF_{1}-aC_{2}\right) \end{array}$$

The local government’s average expected gain is shown in equation (7):


(7) $$\bar{U_{2}}=yU_{21}+\left(1-y\right)U_{22}$$

The replicator dynamics equation for the local government is constructed as follows:


(8) $$\begin{array}{c} \begin{array}{l} F\left(y\right)=\mathrm{dy}/dt=y\left(1-y\right)(p_{1}R_{1}^{\prime }+F_{1}-C_{2}-xp_{1}R_{1}^{\prime }\\ \;-xF_{1}+xzF_{3}+zkF_{3}-xzkF_{3}+m\mu \theta B_{2}-p_{1}R_{1} \end{array}\\ -aF_{1}+aC_{2}+xzmp_{2}Q_{1}+xp_{1}R_{1}+xaF_{1}) \end{array}$$

The central government’s expected gain when it chooses the ‘inspection’ strategy is shown in equation (9):


(9) $$\begin{array}{c} U_{31}=xym\left(v_{1}R_{2}-v_{2}Q_{2}-C_{3}\right)+xy\left(1-m\right)\\ \left(v_{1}R_{2}-v_{2}Q_{2}-C_{3}\right)+x\left(1-y\right)m\left(v_{1}R_{2}-v_{2}Q_{2}-C_{3}\right)\\ +x\left(1-y\right)\left(1-m\right)\left(v_{1}R_{2}-v_{2}Q_{2}-C_{3}\right)+\left(1-x\right)\\ \mathrm{ym}\left(v_{1}R_{1}-vQ2_{1}-C_{3}+F_{2}\right)+\left(1-x\right)y\left(1-m\right)\\ \left(v_{1}R_{1}-v_{2}Q_{1}-C_{3}+F_{2}\right)+\left(1-x\right)\left(1-y\right)\\ m\left(v_{1}R_{1}-v_{2}Q_{1}-C_{3}+kF_{2}\right)+\left(1-x\right)\left(1-y\right)\left(1-m\right)\\ \left(v_{1}R_{1}-v_{2}Q_{1}-C_{3}+kF_{2}\right) \end{array}$$

The central government’s expected gain when it chooses the ‘no inspection’ strategy is shown in equation (10):


(10) $$\begin{array}{l} U_{32}=xym\left(v_{1}R_{2}-v_{2}Q_{2}\right)+xy\left(1-m\right)\left(v_{1}R_{2}-v_{2}Q_{2}\right)\\ \;+x\left(1-y\right)m\left(v_{1}R_{2}-v_{2}Q_{2}\right)+x\left(1-y\right)\left(1-m\right)\\ \;\left(v_{1}R_{2}-v_{2}Q_{2}\right)+\left(1-x\right)\mathrm{ym}\left(v_{1}R_{1}-v_{2}Q_{1}\right)\\ \;+\left(1-x\right)y\left(1-m\right)\left(v_{1}R_{1}-v_{2}Q_{1}\right)+\left(1-x\right)\left(1-y\right)\\ \;m\left(v_{1}R_{1}-v_{2}Q_{1}\right)+\left(1-x\right)\left(1-y\right)\\ \;\left(1-m\right)\left(v_{1}R_{1}-v_{2}Q_{1}\right) \end{array}$$

The central government’s average expected gain is shown in equation (11):


(11) $$\bar{U_{3}}=zU_{31}+\left(1-z\right)U_{32}$$

The replicator dynamics equation of the central government is constructed as follows:


(12) $$\begin{array}{l} F\left(z\right)=dz/dt=z\left(1-z\right)(yF_{2}-xyF_{2}-C_{3}+kF_{2}\\ \;-ykF_{2}-xkF_{2}+\;xykF_{2}) \end{array}$$

The media’s expected gain when it chooses the ‘exposure’ strategy is shown in equation (13):


(13) $$\begin{array}{l} \begin{array}{l} U_{41}=xyz\left(G-C_{4}\right)+xy\left(1-z\right)\left(G-C_{4}\right)+x\left(1-y\right)\\ \;z\left(G-C_{4}+H\right)+x\left(1-y\right)\left(1-z\right)\left(G-C_{4}\right)+\left(1-x\right) \end{array}\\ \begin{array}{l} \;yz\left(G-C_{4}+H\right)+\left(1-x\right)y\left(1-z\right)\left(G-C_{4}\right)+\left(1-x\right)\\ \;\left(1-y\right)z\left(G-C_{4}+H\right)+\left(1-x\right)\left(1-y\right)\left(1-z\right)\left(G-C_{4}\right)\\ \; \end{array} \end{array}$$

The media’s expected gain when it chooses the ‘nonexposure’ strategy is shown in equation (14):


(14) $$U_{42}=0$$

The average expected gain of the media is shown in equation (15):


(15) $$\bar{U_{4}}=mU_{41}+\left(1-m\right)U_{42}$$

The replicator dynamics equation of the media is constructed as follows:


(16) $$F\left(m\right)=\mathrm{dm}/dt=m\left(1-m\right)\left(G-C_{4}-\mathrm{xyzH}+zH\right)$$

Combining Equations (4), (8), (12), and (16), the evolutionary game replicator dynamics system of the enterprise, local government, central government and media can be obtained as shown in equation (17):


(17) $$\left\{ \begin{array}{c} F\left(x\right)=x\left(1-x\right)(R_{2}-\alpha Q_{2}+\alpha \beta Q_{2}-C_{1}+yzF_{2}+yF_{1}\\ +zkF_{2}-yzkF_{2}+m\mu \theta B_{1}-R_{1}+\alpha Q_{1}+aF_{1}-yaF_{1})\\ F\left(y\right)=y\left(1-y\right)(p_{1}R_{1}^{\prime }+F_{1}-C_{2}-xp_{1}R_{1}^{\prime }-xF_{1}+xzF_{3}\\ +zkF_{3}-xzkF_{3}+m\mu \theta B_{2}-p_{1}R_{1}-aF_{1}\\ +aC_{2}+xzmp_{2}Q_{1}+xp_{1}R_{1}+xaF_{1})\\ F\left(z\right)=z\left(1-z\right)(yF_{2}-xyF_{2}-C_{3}+kF_{2}-ykF_{2}\\ -xkF_{2}+xykF_{2})\\ F\left(m\right)=m\left(1-m\right)\left(G-C_{4}-\mathrm{xyzH}+zH\right) \end{array}\right.$$

### 3.4 Model analysis

#### 3.4.1 Enterprise strategic stability analysis

First, we take the first partial derivative of $F\left(x\right)$ with respect to x and set $F^{\prime }\left(x\right)=0$ to obtain: $z^{*}=\frac{-R_{2}+\alpha Q_{2}-\alpha \beta Q_{2}+C_{1}-yF_{1}-m\mu \theta B_{1}+R_{1}-\alpha Q_{1}-aF_{1}+yaF_{1}}{yF_{2}+kF_{2}-ykF_{2}}$ . When $z=z^{*}$ , $0<z^{*}<1$ , then $F\left(x\right)\text{≡}0$ ; at this time, regardless of the value of x , the enterprise is in a stable state, and the choice of strategy does not change over time. If $z\neq z^{*}$ , substituting x=0 , x=1 into $F^{\prime }\left(x\right)$ yields: (1) When $0<z<z^{*}<1$ , then: $F^{\prime }\left(x\right)|{}_{x=0}<0$ , $F^{\prime }\left(x\right)|{}_{x=1}>0$ , and at this time, x=0 is a systematic evolutionary stable strategy; that is, the enterprise chooses the strategy of “no pollution control”. (2) When $0<z^{*}<z<1$ , then: $F^{\prime }\left(x\right)|{}_{x=0}>0$ , $F^{\prime }\left(x\right)|{}_{x=1}<0$ , and at this time, x=1 is a systematic evolutionary stable strategy; that is, the enterprise chooses the “pollution control” strategy.

Based on the enterprise strategic stability analysis above, the following inferences can be obtained:

*Inference 1*: The choice of behavioral strategy of the enterprise is influenced by the behavioral strategy of the central government. When the probability of the central government choosing the “inspection” strategy is low, the enterprise is inclined to choose the “no pollution control” option; when the probability of the central government choosing “inspection” is high, enterprises tend to choose the “pollution control” option. Therefore, inspection by the central government is conducive to promoting the pollution control behavior of enterprises.

*Inference 2*: The probability of the enterprise choosing “pollution control” is positively correlated with the income difference between the enterprise’s pollution control and the nonpollution control period $R_{2}-R_{1}$ and negatively correlated with the pollution control cost of the enterprise $C_{1}$ . That is, the larger the income difference between pollution control and nonpollution control is, and the lower the enterprise’s emission reduction cost is, the higher the probability of the enterprise choosing the “pollution control” strategy.

*Inference 3*: The probability of enterprises choosing “pollution control” is positively correlated with the proportion of tax reductions or exemptions implemented by local governments for pollution control enterprises β , the environmental protection tax rate α , the extent to which local governments implement environmental policies a , and the penalties imposed by local governments on the illegal pollutant discharge enterprises $F_{1}$ . That is, the higher the tax reduction ratio implemented by the local government to the pollution control enterprises is, the higher the environmental protection tax rate is, the greater the implementation degree of the environmental policies of local governments is, and the heavier the penalties that local governments impose on violating enterprises is, the more inclined that enterprises are to choose the “pollution control” strategy.

*Inference 4*: The larger that the central government’s penalty on the violating enterprise $F_{2}$ and penalty multiple k are, the more inclined the enterprise is to choose the “pollution control” strategy.

*Inference 5*: The larger the reputation loss caused by media exposure to the enterprise $B_{1}$ is, the larger the probability that the media successfully discovers regulatory capture μ , the greater the influence of media exposure θ , and the higher the probability of the enterprise choosing the “pollution control” strategy.

The inferences above show that the larger the gap between the gains obtained by enterprises from pollution control and emission reduction and those obtained from no pollution control or less pollution control is, the more likely that enterprises are to choose pollution control. Therefore, the government should actively encourage and support the green technology development of enterprises and adopt incentives such as subsidies and rewards for R&D and the purchase of advanced equipment to promote corporate pollution control and emission reduction. The active implementation of environmental policies by local governments, the inspection and punishment mechanism of the central government and media exposure all have a certain inhibitory effect on the illegal pollutant discharge behaviors of enterprises. In terms of policy mix, local governments can combine the environmental protection tax rate and the environmental protection tax reduction ratio to increase the constraining effect of both positive and negative aspects on the enterprise. In addition, attention should be given to the independent supervisory role of the media, which can effectively supplement the deficiencies in the activities of government supervision agencies.

#### 3.4.2 Analysis of local government strategic stability

We take the first partial derivative of $F\left(y\right)$ with respect to y , and set $F^{\prime }\left(y\right)=0$ to obtain: $z^{*}=\frac{-p_{1}R_{1}^{\prime }-F_{1}+C_{2}+xp_{1}R_{1}^{\prime }+xF_{1}-m\mu \theta B_{2}+p_{1}R_{1}+aF_{2}-aC_{2}-xp_{1}R_{1}-xaF_{1}}{xF_{3}+kF_{3}-xkF_{3}+xmp_{2}Q_{1}}$ . When $z=z^{*}$ , $0<z^{*}<1$ , then $F\left(y\right)\text{≡}0$ ; at this time, regardless of the value of y , the local government is in a stable state, and the choice of strategy does not change over time. If $z\neq z^{*}$ , substituting y=0 , y=1 into $F^{\prime }\left(y\right)$ , yields: (1) When $0<z<z^{*}<1$ , then: $F^{\prime }\left(y\right)|{}_{y=0}<0$ , $F^{\prime }\left(y\right)|{}_{y=1}>0$ , at this time y=0 is a systematic evolutionary stable strategy; that is, the local government chooses the “passive implementation” strategy. (2) When $0<z^{*}<z<1$ , then: $F^{\prime }\left(y\right)|{}_{y=0}>0$ , $F^{\prime }\left(y\right)|{}_{y=1}<0$ , at this time y=1 is a systematic evolutionary stable strategy; that is, the local government chooses the “active implementation” strategy.

Based on the analysis of local government strategic stability above, the following inferences can be drawn:

*Inference 6*: The selection of behavioral strategies of the local government is influenced by the behavioral strategy of the central government. When the probability of the central government choosing the “inspection” strategy is below a certain threshold, the local government is inclined to choose “passive implementation.” When the probability of the central government choosing the “inspection” strategy is high, local governments tend to choose “active implementation.” Therefore, central government inspection is conducive to the active implementation of environmental policies and the performance of regulatory functions by local governments.

*Inference 7*: The higher the gains that the enterprise receives from not controlling its pollution $R_{1}$ are, the more taxes enterprises pay to local governments, and the higher the probability of local governments choosing to passively implement environmental policies, and the more readily that local governments are captured by regulation.

*Inference 8*: The probability that the local government chooses the “active implementation” strategy is negatively correlated to the cost of the local government’s active implementation of environmental policies $C_{2}$ and the economic preference of the local government $p_{1}$ , while it is positively correlated with the level of fines imposed by local governments on enterprises that do not control their pollution $F_{1}$ and the environmental preference of local governments $p_{2}$ . In other words, when the cost of active implementation of environmental policies for the local government is lower, the degree of economic preference is also lower, the fines for enterprises not controlling their pollution are higher, and the environmental preference is higher. Therefore, local governments are more inclined to choose the “active implementation” strategy.

*Inference 9*: When the central government imposes greater political penalties $F_{3}$ and penalty multiplier k on local governments for failure to implement environmental policies, local governments are more inclined to choose the “active implementation” strategy and less likely to be captured.

*Inference 10*: The greater the local government’s reputation loss caused by media exposure $B_{2}$ is, the greater the probability that the media successfully discovers regulatory capture μ , and the greater the influence of media exposure θ . The probability of the local government choosing the “active implementation” strategy is higher, and the probability of being captured is reduced.

The inferences above show that when enterprises contribute more to their local economy, the probability of local governments being captured is higher. Compared with small enterprises, local governments are more likely to be captured by large local enterprises, resulting in policies that are biased toward large enterprises and are contrary to the policies of the central government. The economic preferences and environmental preferences of local governments have a great influence on their own behavioral choices. These two preferences are mainly affected by the performance appraisal policy of the central government. Therefore, for the central government, it is necessary to develop reasonable performance appraisal standards for local government performance. To develop assessment criteria, the economy and the environment must be considered simultaneously. Active inspection by the central government can promote the active implementation of environmental policies by local governments and the pollution control of enterprises, thus helping to overcome the regulatory capture that occurs between the local government and enterprises. In addition, media supervision and exposure exert adverse negative impacts on local governments that passively implement environmental policies, leading to reputation loss, such as a decline in local governments’ social credibility. This forces local governments to actively implement environmental governance policies and preventing regulatory capture.

#### 3.4.3 The central government’s strategic stability

We take the first partial derivative of $F\left(z\right)$ with respect to z _,_ and set $F^{\prime }\left(z\right)=0$ to obtain: $x^{*}=\frac{-yF_{2}+C_{3}-kF_{2}+ykF_{2}}{-yF_{2}-kF_{2}+ykF_{2}}$ . When $x=x^{*}$ , $0<x^{*}<1$ then $F\left(z\right)\text{≡}0$ ; at this time, regardless of the value of z , the central government is in a stable state, and the choice of strategy does not change over time. If $x\neq x^{*}$ , substituting z=0 , z=1 into $F^{\prime }\left(z\right)$ , yields: (1) When $0<x<x^{*}<1$ , then: $F^{\prime }\left(z\right)|{}_{z=0}>0$ , $F^{\prime }\left(z\right)|{}_{z=1}<0$ , at this time z=1 is a systematic evolutionary stable strategy; that is, the central government chooses the “inspection” strategy. (2) When $0<x^{*}<x<1$ , then: $F^{\prime }\left(z\right)|{}_{z=0}<0$ , $F^{\prime }\left(z\right)|{}_{z=1}>0$ , at this time z=0 is a systematic evolutionary stable strategy; that is, the central government chooses the “no inspection” strategy.

Based on the analysis of the central government’s strategic stability above, the following inferences can be drawn:

*Inference 11*: The central government’s choice of behavioral strategies is influenced by the behavioral strategy of enterprises. When the probability of enterprises choosing the “pollution control” strategy is high, the central government is inclined to choose “no inspection” option. Conversely, when the enterprise chooses the “pollution control” strategy with a low probability, the central government is inclined to choose the “inspection” option.

*Inference 12*: The probability of the central government choosing the “inspection” option is positively correlated with the fines the central government imposes on violating enterprises $F_{2}$ and negatively correlated with the inspection cost of the central government $C_{3}$ ; that is, the higher the fine the central government imposes on violating enterprises that do not control their pollution is, the lower the inspection cost, and the more inclined the central government is to choose the “inspection” strategy.

The inferences above indicate that the central government needs to comprehensively consider economic development and environmental protection when making decisions, but its final selection of strategy is only affected by the inspection costs and penalty amounts imposed on violating enterprises. The penalties for violating enterprises, on the one hand, help to constrain the behavior of enterprises and encourage them to adopt pollution control behaviors. On the other hand, they can reduce the inspection costs of the central government and increase the enthusiasm for inspection, which is ultimately conducive to the prevention of regulatory capture.

#### 3.4.4 Strategic stability of the media

We take the first partial derivative of $F\left(m\right)$ with respect to m and set $F^{\prime }\left(m\right)=0$ to obtain $x^{*}=\frac{G-C_{4}+zH}{yzH}$ . When $x=x^{*}$ , $0<x^{*}<1$ , then $F^{\prime }\left(m\right)\text{≡}0$ ; at this time, regardless of the value of m , the media has a steady state, and the selection of strategy does not change over time. If $x\neq x^{*}$ , substituting m=0 , m=1 into $F^{\prime }\left(m\right)$ , yields: (1) When $0<m<m^{*}<1$ , then: $F^{\prime }\left(m\right)|{}_{m=0}>0$ , $F^{\prime }\left(m\right)|{}_{m=0}<0$ , at this time m=1 is a systematic evolutionary stable strategy; that is, the media chooses the “exposure” strategy. (2) When $0<x^{*}<x<1$ , then: $F^{\prime }\left(m\right)|{}_{m=0}<0$ , $F^{\prime }\left(m\right)|{}_{m=1}>0$ , at this time m=0 is a systematic evolutionary stable strategy; that is, the media chooses the “nonexposure” strategy.

Based on the analysis of the media’s strategic stability above, the following inferences can be drawn:

*Inference 13*: The selection of media behavior strategies is affected by the enterprise’s behavior strategy. When the probability of the enterprise choosing the “pollution control” strategy is high, the media tends to choose “nonexposure.” When the probability of the enterprise choosing the “pollution control” strategy is low, the media tends to choose “exposure.”

*Inference 14*: The probability of the media choosing “exposure” is positively correlated with the level of public funding G and rewards from the central government to the media H and negatively correlated with the supervision and exposure cost of the media $C_{4}$ ; that is, the more public supports for the media that there is, the more the central government rewards the media, the lower the supervision and exposure cost of the media, and the more inclined the media is to choose the “exposure” strategy.

The inferences above show that media supervision exerts a certain inhibitory effect on the regulatory capture of enterprises and local governments, and whether the media adopts the behavior of supervision and exposure mainly depends on the net gains of the media. Incentive measures such as public funding and central government rewards can effectively stimulate the media to play the role of supervision. Therefore, the rational use of the media’s role as a powerful assistant to the regulatory department can effectively inhibit the occurrence of regulatory capture.

### 3.5 Stability analysis of the equilibrium point of the four-party evolutionary game system

We order $F\left(x\right)=0$ , $F\left(y\right)=0$ , $F\left(z\right)=0$ , $F\left(m\right)=0$ , and obtain 16 pure-strategy equilibrium points as follows: $K_{1}\left(1,1,1,1\right)$ , $K_{2}\left(1,1,1,0\right)$ , $K_{3}\left(1,1,0,1\right)$ , $K_{4}\left(1,1,0,0\right)$ , $K_{5}\left(1,0,1,1\right)$ , $K_{6}\left(1,0,1,0\right)$ , $K_{7}\left(1,0,0,1\right)$ , $K_{8}\left(1,0,0,0\right)$ , $K_{9}\left(0,1,1,1\right)$ , $K_{10}\left(0,1,1,0\right)$ , $K_{11}\left(0,1,0,1\right)$ , $K_{12}\left(0,1,0,0\right)$ , $K_{13}\left(0,0,1,1\right)$ , $K_{14}\left(0,0,1,0\right)$ , $K_{15}\left(0,0,0,1\right)$ and $K_{16}\left(0,0,0,0\right)$ . According to the method proposed by Friedman (90), the stability of the 16 equilibrium points is determined by analyzing the local stability of the Jacobian matrix of the differential equation system. The corresponding Jacobian matrix is shown as follows:


$$J=\left[\begin{array}{cccc} J_{11} & J_{12} & J_{13} & J_{14}\\ J_{21} & J_{22} & J_{23} & J_{24}\\ J_{31} & J_{32} & J_{33} & J_{34}\\ J_{41} & J_{42} & J_{43} & J_{44} \end{array}\right]=\left[\begin{array}{cccc} \frac{\partial F(x)}{\partial x} & \frac{\partial F(x)}{\partial y} & \frac{\partial F(x)}{\partial z} & \frac{\partial F(x)}{\partial m}\\ \frac{\partial F(y)}{\partial x} & \frac{\partial F(y)}{\partial y} & \frac{\partial F(y)}{\partial z} & \frac{\partial F(y)}{\partial m}\\ \frac{\partial F(z)}{\partial x} & \frac{\partial F(z)}{\partial y} & \frac{\partial F(z)}{\partial z} & \frac{\partial F(z)}{\partial m}\\ \frac{\partial F(m)}{\partial x} & \frac{\partial F(m)}{\partial y} & \frac{\partial F(m)}{\partial z} & \frac{\partial F(m)}{\partial m} \end{array}\right]$$

where:


$$\begin{array}{l} J_{11}=\left(1-2x\right)R_{2}-\alpha Q_{2}+\alpha \beta Q_{2}-C_{1}+yzF_{2}+yF_{1}+zkF_{2}\\ \;\;\;\;\;\;\;\;\;\;-\;yzkF_{2}+m\mu \theta B_{1}-R_{1}+\alpha Q_{1}+aF_{1}-yaF_{1}) \end{array}$$

$$J_{12}=x\left(1-x\right)\left(zF_{2}+F_{1}-zkF_{2}-aF_{1}\right)$$

$$J_{13}=x\left(1-x\right)\left(yF_{2}+kF_{2}-ykF_{2}\right)$$

$$J_{14}=x\left(1-x\right)\times \mu \theta B_{1}$$

$$J_{21}=y\left(1-y\right)\left(-p_{1}R_{1}^{\prime }-F_{1}+zF_{3}-zkF_{3}+zmp_{2}Q_{1}+p_{1}R_{1}+aF_{1}\right)$$

$\begin{array}{l} J_{22}=\left(1-2y\right)(p_{1}R_{1}^{\prime }+F_{1}-C_{2}-xp_{1}R_{1}^{\prime }-xF_{1}+xzF_{3}\\ \;\;\;\;\;\;\;\;\;\;+zkF_{3}-xzkF_{3}+m\mu \theta B_{2}-p_{1}R_{1}-aF_{1}+aC_{2}\\ \;\;\;\;\;\;\;\;\;\;+xzmp_{2}Q_{1}+xp_{1}R_{1}+xaF)\\ J_{23}=y\left(1-y\right)\left(xF_{3}+kF_{3}-xkF_{3}+xmp_{2}Q_{1}\right) \end{array}$


$$J_{24}=y\left(1-y\right)\left(\mu \theta B_{2}+xzp_{2}Q_{1}\right)$$

$$J_{31}=z\left(1-z\right)\left(-yF_{2}-kF_{2}+ykF_{2}\right)$$

$$J_{32}=z\left(1-z\right)\left(F_{2}-xF_{2}-kF_{2}+xkF_{2}\right)$$

$$J_{33}=\left(1-2z\right)\left(yF_{2}-xyF_{2}-C_{3}+kF_{2}-ykF_{2}-xkF_{2}+xykF_{2}\right)$$

$$J_{34}=0$$

$$J_{41}=m\left(1-m\right)\left(-yzH\right)$$

$$J_{42}=m\left(1-m\right)\left(-xzH\right)$$

$$J_{43}=m\left(1-m\right)\left(-xyH+H\right)$$

$$J_{44}=\left(1-2m\right)\left(G-C_{4}-xyH+zH\right)$$

According to the Lyapunov stability determination rule, when all the eigenvalues of the Jacobian matrix are negative real numbers, that is, when $\lambda_{1}<0$ , $\lambda_{2}<0$ , $\lambda_{3}<0$ and $\lambda_{4}<0$ are simultaneously satisfied, the corresponding equilibrium point is a stable point; when at least one eigenvalue of the Jacobian matrix is a positive real number, the corresponding equilibrium point is a saddle point; and when the eigenvalues of the Jacobian matrix are all positive real numbers, the corresponding equilibrium point is an unstable point. Substituting the 16 partial equilibrium points into the Jacobian matrix, and based on the assumptions above, we obtain the eigenvalues corresponding to each equilibrium point, as shown in Table 3.

**Table 3:** Eigenvalues of partial equilibrium points.

Equilibrium point	$\lambda_{1}$	$\lambda_{2}$	$\lambda_{3}$	$\lambda_{4}$
$K_{1}\left(1\text{,}1\text{,}1\text{,}1\right)$	$R_{1}-R_{2}+\left(1-\beta \right)\alpha Q_{2}-\alpha Q_{1}+C_{1}-F_{1}-F_{2}-\mu \theta B_{1}$	$\left(1-a\right)C_{2}-F_{3}-\mu \theta B_{2}-p_{2}Q_{1}$	$C_{3}$	$C_{4}-G$
$K_{2}\left(1\text{,}1\text{,}1\text{,}0\right)$	$R_{1}-R_{2}+\left(1-\beta \right)\alpha Q_{2}-\alpha Q_{1}+C_{1}-F_{1}-F_{2}$	$\left(1-a\right)C_{2}-F_{3}$	$C_{3}$	$G-C_{4}$
$K_{3}\left(1\text{,}1\text{,}0\text{,}1\right)$	$R_{1}-R_{2}+\left(1-\beta \right)\alpha Q_{2}-\alpha Q_{1}+C_{1}-F_{1}-\mu \theta B_{1}$	$\left(1-a\right)C_{2}-\mu \theta B_{2}$	$-C_{3}$	$C_{4}-G$
$K_{4}\left(1\text{,}1\text{,}0\text{,}0\right)$	$R_{1}-R_{2}+\left(1-\beta \right)\alpha Q_{2}-\alpha Q_{1}+C_{1}-F_{1}$	$\left(1-a\right)C_{2}$	$-C_{3}$	$G-C_{4}$
$K_{5}\left(1\text{,}0\text{,}1\text{,}1\right)$	$R_{1}-R_{2}+\left(1-\beta \right)\alpha Q_{2}-\alpha Q_{1}+C_{1}-aF_{1}-kF_{2}-\mu \theta B_{1}$	$-\left(1-a\right)C_{2}+F_{3}+\mu \theta B_{2}+p_{2}Q_{1}$	$C_{3}$	$C_{4}-G-H$
$K_{6}\left(1\text{,}0\text{,}1\text{,}0\right)$	$R_{1}-R_{2}+\left(1-\beta \right)\alpha Q_{2}-\alpha Q_{1}+C_{1}-aF_{1}-kF_{2}$	$-\left(1-a\right)C_{2}+F_{3}$	$C_{3}$	$G+H-C_{4}$
$K_{7}\left(1\text{,}0\text{,}0\text{,}1\right)$	$R_{1}-R_{2}+\left(1-\beta \right)\alpha Q_{2}-\alpha Q_{1}+C_{1}-aF_{1}-\mu \theta B_{1}$	$-\left(1-a\right)C_{2}+\mu \theta B_{2}$	$-C_{3}$	$C_{4}-G$
$K_{8}\left(1\text{,}0\text{,}0\text{,}0\right)$	$R_{1}-R_{2}+\left(1-\beta \right)\alpha Q_{2}-\alpha Q_{1}+C_{1}-aF_{1}$	$-\left(1-a\right)C_{2}$	$-C_{3}$	$G-C_{4}$
$K_{9}\left(0\text{,}1\text{,}1\text{,}1\right)$	$R_{2}-R_{1}+\alpha Q_{1}-\left(1-\beta \right)\alpha Q_{2}-C_{1}+F_{1}+F_{2}+\mu \theta B_{1}$	$\left(1-a\right)C_{2}+p_{1}R_{1}-p_{1}R_{1}^{\prime }-\left(1-a\right)F_{1}-\mu \theta B_{2}-kF_{3}$	$C_{3}-F_{2}$	$C_{4}-G-H$
$K_{10}\left(0\text{,}1\text{,}1\text{,}0\right)$	$R_{2}-R_{1}+\alpha Q_{1}-\left(1-\beta \right)\alpha Q_{2}-C_{1}+F_{1}+F_{2}$	$\left(1-a\right)C_{2}+p_{1}R_{1}-p_{1}R_{1}^{\prime }-\left(1-a\right)F_{1}-kF_{3}$	$C_{3}-F_{2}$	$G+H-C_{4}$
$K_{11}\left(0\text{,}1\text{,}0\text{,}1\right)$	$R_{2}-R_{1}+\alpha Q_{1}-\left(1-\beta \right)\alpha Q_{2}-C_{1}+F_{1}+\mu \theta B_{1}$	$\left(1-a\right)C_{2}+p_{1}R_{1}-p_{1}R_{1}^{\prime }-\left(1-a\right)F_{1}-\mu \theta B_{2}$	$F_{2}-C_{3}$	$C_{4}-G$
$K_{12}\left(0\text{,}1\text{,}0\text{,}0\right)$	$R_{2}-R_{1}+\alpha Q_{1}-\left(1-\beta \right)\alpha Q_{2}-C_{1}+F_{1}$	$\left(1-a\right)C_{2}+p_{1}R_{1}-p_{1}R_{1}^{\prime }-\left(1-a\right)F_{1}$	$F_{2}-C_{3}$	$G-C_{4}$
$K_{13}\left(0\text{,}0\text{,}1\text{,}1\right)$	$R_{2}-R_{1}+\alpha Q_{1}-\left(1-\beta \right)\alpha Q_{2}-C_{1}+aF_{1}+kF_{2}+\mu \theta B_{1}$	$p_{1}R_{1}^{\prime }-p_{1}R_{1}-\left(1-a\right)C_{2}+\left(1-a\right)F_{1}+\mu \theta B_{2}+kF_{3}$	$C_{3}-kF_{2}$	$C_{4}-G-H$
$K_{14}\left(0\text{,}0\text{,}1\text{,}0\right)$	$R_{2}-R_{1}+\alpha Q_{1}-\left(1-\beta \right)\alpha Q_{2}-C_{1}+aF_{1}+kF_{2}$	$p_{1}R_{1}^{\prime }-p_{1}R_{1}-\left(1-a\right)C_{2}+\left(1-a\right)F_{1}+kF_{3}$	$C_{3}-kF_{2}$	$G+H-C_{4}$
$K_{15}\left(0\text{,}0\text{,}0\text{,}1\right)$	$R_{2}-R_{1}+\alpha Q_{1}-\left(1-\beta \right)\alpha Q_{2}-C_{1}+aF_{1}+\mu \theta B_{1}$	$p_{1}R_{1}^{\prime }-p_{1}R_{1}-\left(1-a\right)C_{2}+\left(1-a\right)F_{1}+\mu \theta B_{2}$	$kF_{2}-C_{3}$	$C_{4}-G$
$K_{16}\left(0\text{,}0\text{,}0\text{,}0\right)$	$R_{2}-R_{1}+\alpha Q_{1}-\left(1-\beta \right)\alpha Q_{2}-C_{1}+aF_{1}$	$p_{1}R_{1}^{\prime }-p_{1}R_{1}-\left(1-a\right)C_{2}+\left(1-a\right)F_{1}$	$kF_{2}-C_{3}$	$G-C_{4}$

As shown in Table 3, there are 11 possible stability strategy situations in the game system consisting of enterprises, local governments, the central government and the media: $K_{3}\left(1\text{,}1\text{,}0\text{,}1\right)$ , $K_{7}\left(1\text{,}0\text{,}0\text{,}1\right)$ , $K_{8}\left(1\text{,}0\text{,}0\text{,}0\right)$ , $K_{9}\left(0\text{,}1\text{,}1\text{,}1\right)$ , $K_{10}\left(0\text{,}1\text{,}1\text{,}0\right)$ , $K_{11}\left(0\text{,}1\text{,}0\text{,}1\right)$ , $K_{12}\left(0\text{,}1\text{,}0\text{,}0\right)$ , $K_{13}\left(0\text{,}0\text{,}1\text{,}1\right)$ , $K_{14}\left(0\text{,}0\text{,}1\text{,}0\right)$ , $K_{15}\left(0\text{,}0\text{,}0\text{,}1\right)$ and $K_{16}\left(0\text{,}0\text{,}0\text{,}0\right)$ .

Comparing the 11 scenarios above shows that the strategic choices of enterprises, local governments, the central government, and the media are related to the benefits that they receive and the expenditures that they incur. (1) Regardless of whether the local government chooses to actively perform its regulatory function, whether the central government conducts inspection or not, and whether the media exposes or not, as long as the benefits of enterprise pollution control are lower than the gains from maintaining the original production without pollution control, then the enterprise will definitely not choose to conduct pollution control. (2) When the gains from the active implementation of environmental policies by local governments are greater than the gains from the passive implementation of environmental policies, even if the central government does not inspect and the media is not exposed, then local governments will still choose to actively implement environmental policies and perform their regulatory functions. (3) The fundamental purpose of the central government is to maximize the overall welfare of society, and the central government needs to balance the relationship between economic development and environmental protection in its decision making processes, but this does not affect its final behavioral choices. As long as the central government can make up for the cost of the inspection by the fine revenue from the violating enterprises obtained through the inspection, then the central government will conduct inspection. (4) The choice of media strategy depends on the size of the potential gains. When the sum of the social public funding and the central government rewards that the media receives from the exposure is greater than its supervision cost, then the media will choose to expose. (5) With the inspection of the central government and the exposure of the media, the behavioral strategies of enterprises and local governments are more likely to evolve to the ideal state, while in the absence of central government inspection, it is almost impossible to achieve the ideal state. Therefore, inspection by the central government and supervision by the media can effectively prevent the phenomenon of regulatory capture, and inspection by the central government is the key to overcoming the dilemma of regulatory capture.

## 4 Numerical simulation analysis

**Figure 1:**
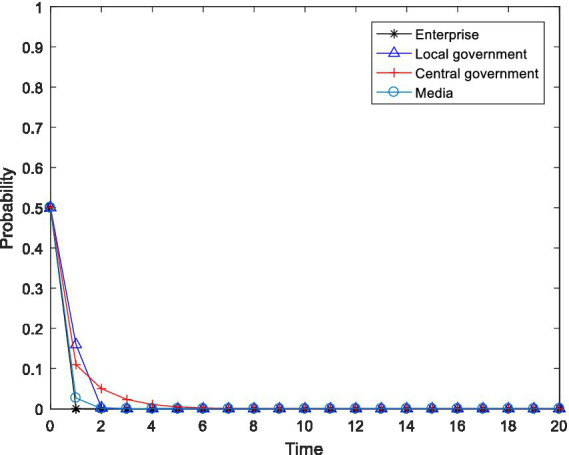
Evolution trajectory of $K_{16}\left(0\text{,}0\text{,}0\text{,}0\right)$ .

From the analysis above, it can be seen that the equilibrium of each stable point can be established under certain conditions. However, considering the practical significance, we conduct further simulation analysis on the least ideal situation, i.e., $K_{16}\left(0\text{,}0\text{,}0\text{,}0\right)$ , explore the effect of key parameters on the evolution of game agent behavior and determine the path from the least ideal state to the ideal state. Based on the replicator dynamics equations and stability conditions above and referring to the parameter settings noted in the literature (6, 10) and the environmental protection tax law, the initial values of the parameters in the present study are set as follows: $R_{1}=30$ , $R_{2}=25$ , $R_{1}^{\prime }=27$ , $B_{1}=5$ , $B_{2}=5$ , $C_{1}=15$ , $C_{2}=10$ , $C_{3}=8$ , $C_{4}=8$ , $p_{1}=0.9$ , $p_{2}=0.1$ , $Q_{1}=12$ , $Q_{2}=9$ , α=1.2 , β=0.25 , μ=0.8 , θ=1.2 , k=1.2 , a=0.5 G=4 , H=2 , $F_{1}=5$ , $F_{2}=6$ , and $F_{3}=10$ . Additionally, the initial probabilities of the four parties are $x_{0}=0.5$ , $y_{0}=0.5$ , $z_{0}=0.5$ and $m_{0}=0.5$ . The simulation software used is MATLAB 2016b, and the simulation result is shown in Figure 1.

As shown in Figure 1, when the initial participation willingness of the enterprise, the local government, the central government and the media were all 0.5, numerical simulation showed that the four-party evolutionary game subject eventually evolved to the undesirable state of x=0 , y=0 , z=0 and m=0 , which is the asymptotic stable point mentioned above $K_{16}\left(0\text{,}0\text{,}0\text{,}0\right)$ . Specifically, when the net gains from pollution control and the emission reduction in enterprises are lower than the difference between the gains obtained from maintaining the original production and the penalties imposed by the local and central governments and the reputation loss caused by the media, the enterprise will choose to not carry out pollution control. Based on the economic preferences of local governments and the performance evaluation criteria of local officials, when the active implementation of environmental policy by local governments would damage local economic development and the utility obtained through active implementation is less than that obtained from passive implementation of environmental policy, then the local government will choose to negatively implement environmental policy. When the fines levied on enterprises by the central government during inspection cannot compensate for its inspection costs, the central government will choose to not conduct inspections, which is unfavorable for regulatory capture. As a rational economic agent, the fundamental purpose of the media is to maximize its own interests. When the gains obtained from media supervision are not enough to cover the supervision costs, the media will not choose the exposure strategy. On this basis, we conduct numerical regulation in this paper on the relevant influencing factors in an attempt to reveal the critical path leading the system’s evolution to the ideal state.

### 4.1 Severity of the penalties imposed by the central government on enterprises and local governments

**Figure 2:**
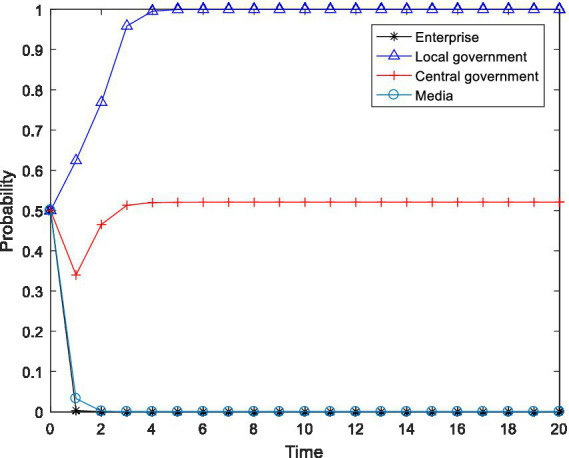
Tetragonal evolution diagram for $F_{2}=8,\;\;F_{3}=12$ .

**Figure 3:**
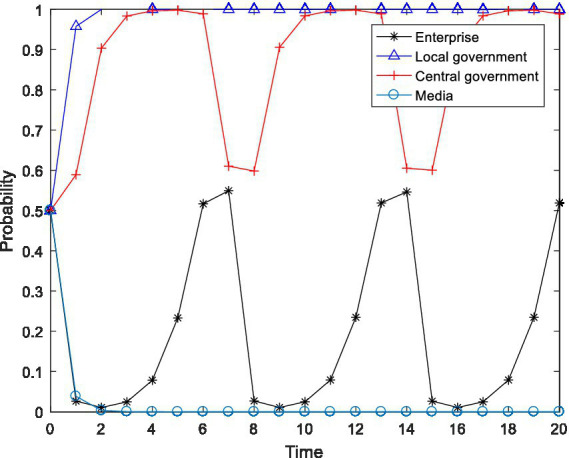
Tetragonal evolution diagram for $F_{2}=10,\;\;F_{3}=14$ .

**Figure 4:**
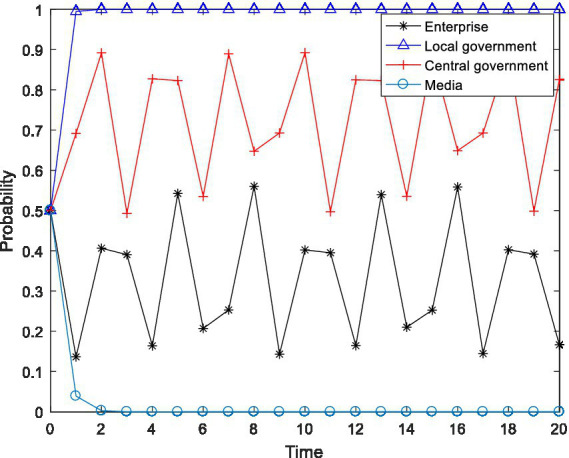
Tetragonal evolution diagram for $F_{2}=12,\;\;F_{3}=16$ .

The constraint mechanism is a common means used by the central government to regulate the behavior of enterprises and local governments. Both enterprises and local governments have bounded rationality and are motivated to seek advantages and avoid disadvantages. Therefore, the penalties imposed by the central government exert certain restraints on the behavior of both parties, and enterprises and local governments can change their behavioral strategies. Keeping other parameters constant, we set ( $F_{2}=8$ , $F_{3}=12$ ), ( $F_{2}=10$ , $F_{3}=14$ ), ( $F_{2}=12$ , $F_{3}=16$ ), and observed the changes in the behavioral choices of the four-party agent, and the results are shown in Figures 2–4.

By comparing Figures 1, 2, it can be seen that as the central government increases the penalties on enterprises and local governments, local governments soon evolve to the stable state of “active implementation,” which can avoid the capture of local governments through regulation and increase the probability of the central government choosing the “inspection” strategy. The reason is that the penalties imposed by the central government will bring greater losses to local governments, including tangible and intangible losses, such as failing performance appraisals of local governments and a decline in social credibility. After weighing these losses, local governments are bound to actively implement environmental policies. Furthermore, a comparison of Figures 2, 4 shows that even if the level of the central government’s penalties is further increased, the behavioral choices of the enterprises do not evolve to a stable state but rather exhibit periodical oscillation. The reason is that the polluting behavior of enterprises is somewhat hidden and is not easily detected by the central government. Thus, for enterprises to gain more revenue, there is a moral hazard. At the same time, the behavior choices of the central government also show a cyclical oscillation state, and the enterprises and the central government are constantly engaged in the game. Therefore, compared to enterprises, local governments are more sensitive to central government penalties.

In summary, through a binding penalty policy, the central government can encourage local governments to actively implement environmental governance policies, effectively prevent local governments from being captured by regulation, and stimulate some enterprises to initiate pollution control.

### 4.2 Environmental protection tax rate

**Figure 5:**
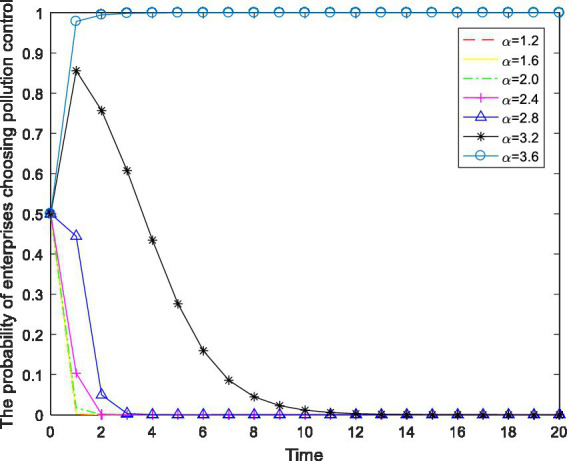
The impact of changes in environmental tax rates α on enterprise behavior.

As an effective policy for promoting enterprise pollution control, environmental protection taxes play an important role in environmental pollution control and protection. Keeping other parameters constant, the environmental tax rate is numerically regulated to observe the changes in enterprise behavioral choices. The results are shown in Figure 5.

As shown in Figure 5, the probability of an enterprise choosing the “pollution control” strategy is positively correlated with the environmental tax rate, and there is a threshold at play. When the environmental tax rate is low, the enterprise does not carry out pollution control; only when the environmental tax rate exceeds a certain threshold does the enterprise choose the “pollution control” strategy. Specifically, when α≤3.2 , the enterprise’s strategic choice stably converges on “no pollution control,” while when α≥3.6 , the behavioral choice of the enterprise shifts from “no pollution control” to “pollution control.” The reason is that a higher environmental protection tax rate compresses the profit margin of the enterprise, reducing the net income of the enterprise and causing it financial difficulties, which motivates the enterprise to carry out pollution control.

In summary, setting a reasonable environmental tax rate can encourage enterprises to actively carry out pollution control and emission reduction.

### 4.3 Local government penalty levels for violators

**Figure 6:**
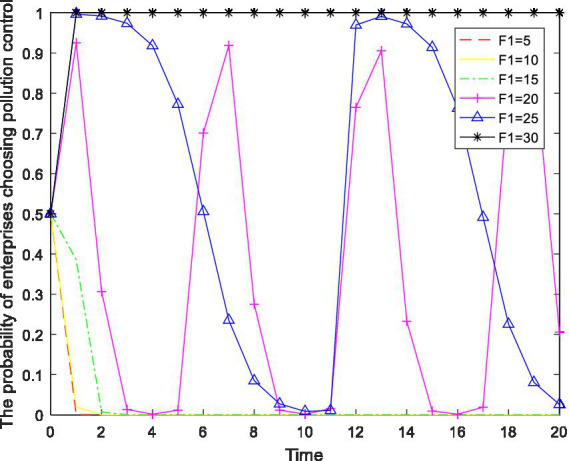
The impact of the changes in *F_1_* on the enterprise’s behavior.

When an enterprise engages in emission violations, it is penalized by the local government. Keeping other parameters constant, the intensity of the that penalty is numerically regulated, and the behavior changes in the enterprise are observed. The results are shown in Figure 6.

As shown in Figure 6, as local governments increase the penalties for violating enterprises, the behavior of such enterprises changes. Specifically, when $0\leq F_{1}\leq 15$ , the enterprise’s behavior stably converges to the strategy of “no pollution control.” When $15<F_{1}<30$ , the behavior of the enterprise changes from “no pollution control” to a cyclical unstable oscillating state. When $F_{1}\geq 30$ , the probability of the enterprise choosing the “pollution control” strategy stably converges at 1. The reason is that when local governments impose light penalties on enterprises, the losses suffered by enterprises from not controlling pollution are relatively small. At this time, enterprises dare to take risks and choose to not control their pollution. Once local governments actively implement environmental policies and institute severe penalties for their violation, it becomes unwise for enterprises to choose the “no pollution control” option. Therefore, the greater the punishment of the local government is, the more the enterprise will be inclined to actively control its pollution. In addition, through a comparison of Figures 4, 6, it can be seen that, compared to the fines levied by the central government, enterprises are more sensitive to the penalties of local governments.

In summary, only when local governments increase the penalty for violating environmental regulations to a certain extent do they encourage enterprises to actively carry out pollution control. At the same time, compared with the fines levied by the central government, the penalties of local governments can more directly and effectively improve the strategic choices of enterprises.

### 4.4 Tax reduction or exemption ratio

**Figure 7:**
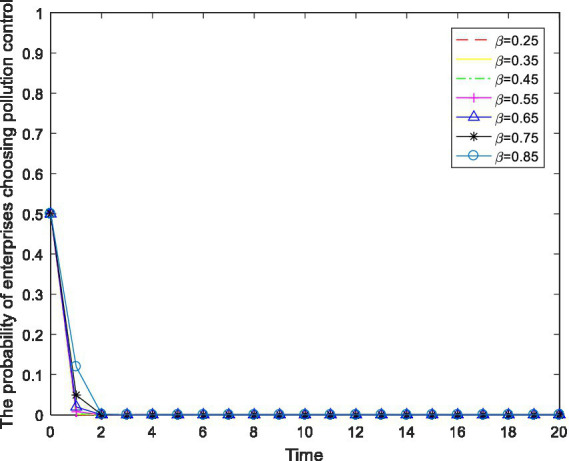
The impact of changes in the tax reduction ratio β on enterprise behavior.

Tax relief is an incentive measure implemented by the government to motivate enterprises to actively carry out pollution control. Keeping other parameters constant, the tax reduction ratio is numerically regulated to observe the enterprise’s behavioral changes. The results are shown in Figure 7.

As shown in Figure 7, for every increase in the tax reduction ratio β of 0.1, the enterprise’s behavioral choice changes very little, and the strategic selection always stably converges at “no pollution control.” The reason is that the net income gained by enterprises from not controlling their pollution is much higher than the income gained by enterprises from controlling their pollution. Furthermore, the tax relief benefit accounts for a relatively small proportion and has little incentive effect on enterprises. Therefore, incentivizing enterprises to control their pollution by merely increasing the proportion of tax relief cannot achieve the desired and ideal effect.

### 4.5 Combined regulation of the environmental protection tax rate and the tax reduction ratio

**Figure 8:**
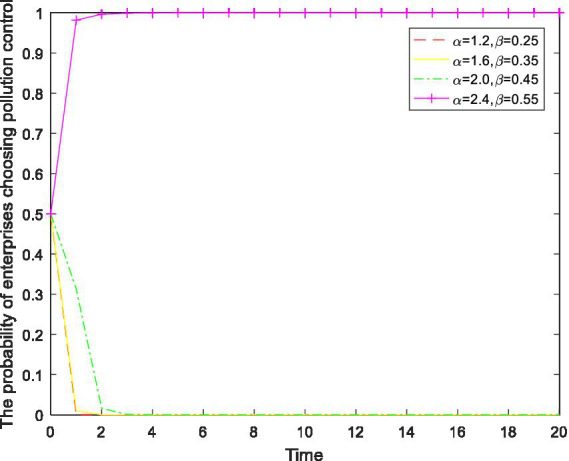
The impact of changes in α and β on enterprise behavior.

It can be seen from the analysis above that solely increasing the environmental protection tax reduction ratio cannot motivate enterprises to control their pollution. Next, we consider the combination of incentive policy and binding policy in the implementation of combined regulation on the environmental protection tax rate and tax reduction ratio to observe the consequent behavioral changes in enterprises. The results are shown in Figure 8.

As shown in Figure 8, as the environmental protection tax rate and the tax reduction ratio simultaneously increase, the behavior of enterprises undergoes a benign change. When α=2.4 , β=0.55 , the strategy choice of the enterprise converges stably to “pollution control” and achieves the ideal expected effect. A comparison of Figures 5, 7, 8 shows that the effect of combining an environmental protection tax policy and a tax relief policy is better than the effect of using an environmental protection tax or a tax relief policy alone. The reason is that, on the one hand, the implementation of an environmental protection tax policy will impose certain constraints on the behavior of enterprises. That is, when enterprises do not treat pollution, they will pay more environmental protection tax, and the corresponding net income of these enterprises will decrease. On the other hand, the implementation of a tax relief policy will have a certain incentive effect on the behavior of enterprises. That is, when enterprises actively control their pollution, they will obtain tax relief concessions from the government, and the corresponding net income of these enterprises will increase. As rational economic persons, enterprises tend to pursue profit and avoid harm. In the case of the implementation of these two policies at the same time, they will inevitably choose the “pollution control” strategy.

In summary, the simultaneous implementation of incentive policies and binding policies can better promote the pollution control behavior of enterprises.

### 4.6 Economic and environmental preferences of local governments

**Figure 9:**
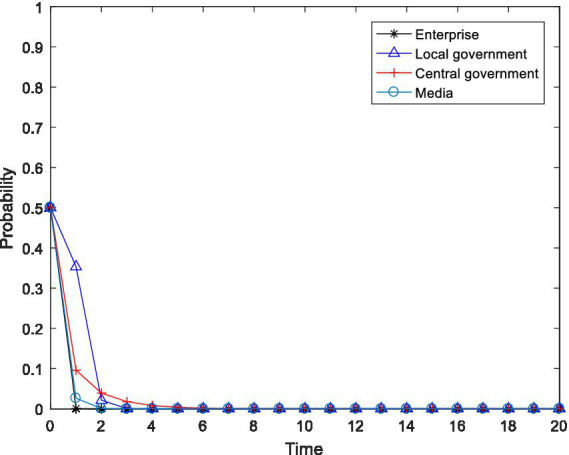
Tetragonal evolution diagram for $p_{1}=0.5,\;\;p_{2}=0.5$ .

**Figure 10:**
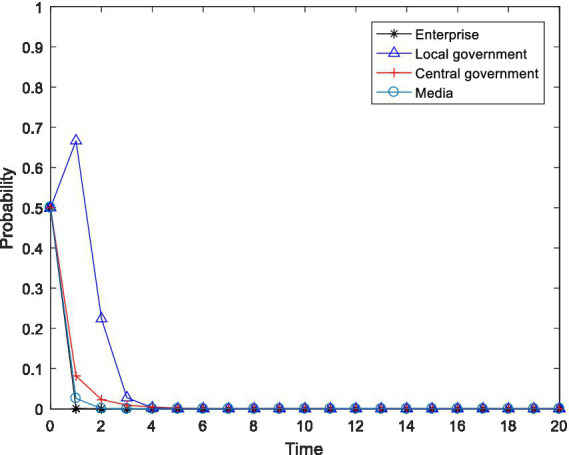
Tetragonal evolution diagram for $p_{1}=0,\;\;p_{2}=0.8$ .

When making decisions, the local government considers local economic development and environmental protection. Keeping other parameters constant, reducing the local government’s economic preference and increasing its environmental preference, $p_{1}=0.5$ , $p_{2}=0.5$ and $p_{1}=0$ , $p_{2}=0.8$ . The results are shown in Figures 9, 10.

**Figure 11:**
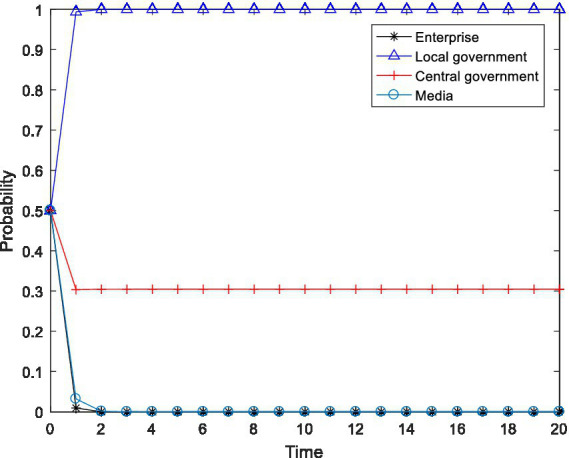
Tetragonal evolution diagram for $p_{1}=0.5,\;\;p_{2}=0.5,\;\;F_{3}=15,\;\;k=2.4$ .

By comparing Figures 1, 9, 10, it can be seen that when reducing the economic preferences of local governments and increasing their environmental preferences, the increase in the willingness of local governments to actively implement environmental policies is relatively small. Even if the economic preferences are reduced to 0, local governments will eventually still be captured by enterprises, putting the system in a poor state. This is due to the lack of a punishment mechanism for local governments. Next, we consider increasing the penalty multiples of the central government imposed on captured local governments so that $p_{1}=0.5$ , $p_{2}=0.5$ , $F_{3}=15$ and k=2.4 , and the results are shown in Figure 11.

As shown in Figure 11, on the basis of reducing the economic preference of local governments and increasing their environmental preferences, when the central government increases the penalty level on local governments, the strategy of local governments stably converges on “active implementation,” the system jumps out of the undesirable state, and the desired effect is achieved. The reason is that improving the assessment index system for local governments alone cannot completely avoid the negative implementation of environmental policies by local governments. It is also necessary for the central government to closely correlate the assessment with punishment to produce strong constraints on the behavior of local governments.

In summary, on the basis of decreasing local governments’ economic preferences and increasing their environmental preferences, strengthening assessment and punishment can encourage local governments to actively implement environmental policies and avoid capture.

### 4.7 Public funding for the media

**Figure 12:**
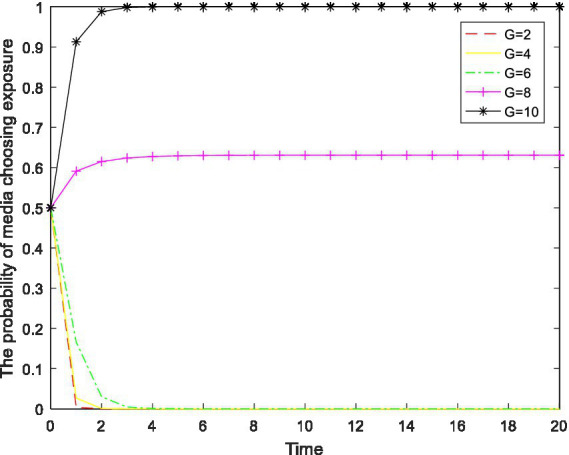
The impact of changes in G on media behavior.

The main purpose of the media, as a rational economic agent, is to maximize its own interests. Therefore, the level of public funding for the media affects its behavior. Keeping other parameters constant, the funding of the public is regulated, and changes in media behavior are observed. The results are shown in Figure 12.

As shown in Figure 12, when the public funding for the media increases, the behavior of the media changes. Specifically, when G≤6 , the media strategy stably converges on “nonexposure”; when 6<G≤8 , the probability of the media choosing the “exposure” strategy is greatly increased, but it cannot converge on 1; and when G≥10 , the media’s strategy stably converges on “exposure.” The reason is that media supervision itself has a certain cost. When the public funding is greater, the benefits of media supervision will also be greater. Therefore, the media’s enthusiasm for supervising will also be improved.

In summary, increasing public funding is conducive to improving the strategic choices of the media.

### 4.8 The impact of media exposure and the reputational losses to enterprises and local governments

**Figure 13:**
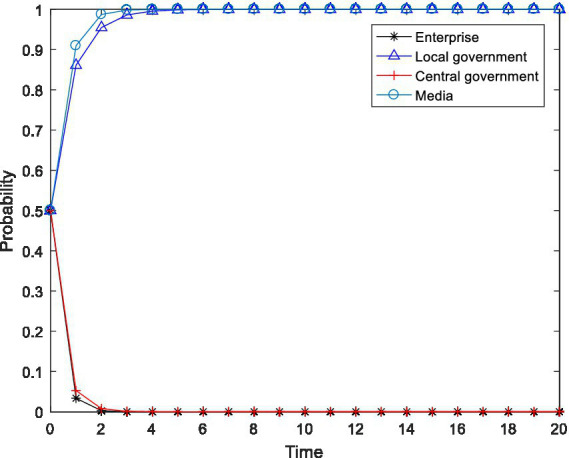
Tetragonal evolution diagram for $\theta =1.6,\;\;B_{1}=5,\;\;B_{2}=5$ .

**Figure 14:**
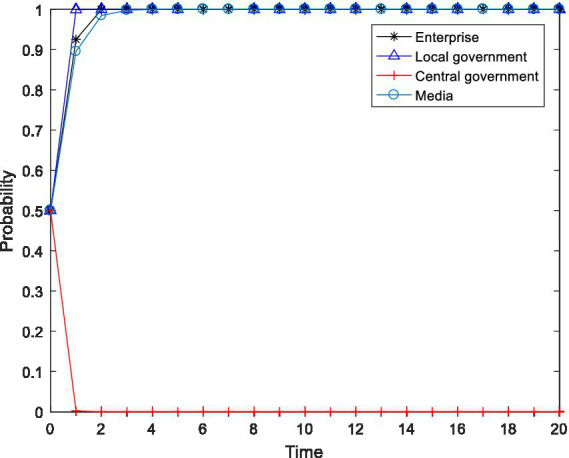
Tetragonal evolution diagram for $\theta =1.8,B_{1}=10,B_{2}=10$ .

Compared with government supervision, media supervision based on public participation has the advantages of rapid dissemination and a wide audience, and it can leveraged to intervene and play a role in the supervision of environmental pollution control. Based on the previous analysis, the level of public funding for the media is set to 10, and the other parameters are kept constant. Through numerical control of the influence of media exposure and the resulting reputation loss to enterprises and local governments, θ=1.6 , $B_{1}=5$ , $B_{2}=5$ and θ=1.8 , $B_{1}=10$ , $B_{2}=10$ , respectively, and the results are shown in Figures 13, 14.

By comparing Figures 1, 13, it can be seen that when the influence of media exposure increases to 1.6, the behavioral choices of enterprises still stably converge on “no pollution control,” while the behavior of local governments changes from “passive implementation” to “active implementation.” Therefore, media supervision can effectively prevent local governments from being captured. Furthermore, the influence of media exposure and the resulting reputation loss to enterprises and local governments should continue to be increased. As shown in Figure 14, when θ=1.8 , $B_{1}=10$ and $B_{2}=10$ , the behavior of enterprises stably converges on “pollution control,” the behavior of local governments stably converges on “active implementation,” the system evolves to the ideal state, and the expected effect is achieved. The reason is that when the influence of media exposure is large, the exposure of the violations of enterprises and local governments will bring enormous losses to both, including tangible and intangible losses, which will effectively restrain the behavior of enterprises and local governments. In addition, a comparison of Figures 2, 11, 14, shows that, compared to the central government’s supervision, media supervision has a better effect on promoting enterprise pollution control and avoiding regulation capture. The reason is that media supervision can more quickly and sensitively detect corporate violations and draw social attention to them, prompting local governments to actively improve corporate environmental behavior.

In summary, the greater the influence of media exposure and the greater the reputation loss to the enterprise and local government is, the greater the degree to which the enterprise actively conducts pollution control, thus the local government is not captured and actively implements environmental policies. Therefore, media supervision can effectively overcome the regulatory capture dilemma caused by the policy burden, and the effect of media supervision is better than that of central environmental inspection.

## 5 Conclusions and suggestions

Based on the assumption of bounded rationality, this study aims to solve the problem of regulatory capture in the governance of environmental pollution in China. It takes central environmental protection inspection and media supervision as a breakthrough, constructs a four-party evolutionary game model consisting of enterprises, local governments, the central government, and the media, analyzes the strategic stability of the four main bodies and the influencing factors, and conducts numerical simulation by using MATLAB 2016b software to analyze the important paths for overcoming regulatory capture. The main conclusions of this study are as follows:

1. Local governments tend to be more concerned about economic development, and large enterprises have a very important role in local economic development. Thus, local governments will formulate policies in favor of large enterprises, reduce the pollution penalties for large enterprises or even not penalize them, thus falling into a dilemma of regulatory capture.
2. Only reducing the economic preferences of local governments without considering the corresponding penalties cannot avoid the negative environmental behaviors of local governments. It is necessary to increase the penalties for the negative environmental behaviors of local governments under the premise of reducing the economic preferences of local governments to motivate local governments to adopt positive environmental behaviors.
3. Compared with the central government, local governments not only impose administrative penalties on noncompliant enterprises but also bring potential losses in terms of environmental credit evaluation, policy preferences, etc. Therefore, local government penalties can more directly and effectively improve the strategic choices of enterprises, prompting them to actively control their pollution.
4. It is difficult to quickly and effectively improve the environmental behavior of enterprises by only increasing the strength of tax relief or the environmental protection tax rate. However, the combination of environmental protection tax and tax relief policies can effectively guide enterprises to actively control their pollution and achieve better results than when only one or the other of the two policies is used.
5. Both central environmental protection inspection and media supervision can effectively improve the environmental behavior of local governments and enterprises. The greater the influence of media exposure is, the stronger the constraints on local governments and enterprises. Compared with central environmental protection inspection, media supervision can more quickly and sensitively detect the violation behaviors of enterprises and prompt local governments to actively implement environmental policies.

Based on the conclusions above, the following policy suggestions are proposed:

1. The performance evaluation system for local governments should be improved to balance economic development and environmental protection. In terms of the assessment mechanism, more quantifiable indicators for environmental protection, pollution remediation and resource conservation should be incorporated into the performance appraisal system for local governments to change the performance evaluation criteria, which are mainly based on economic growth.
2. At the cognitive level, the central government should actively guide local government officials to change their traditional development concepts, and it should vigorously publicize new development concepts to fundamentally change local governments’ understanding of environmental protection. At the same time, the central government should improve the mechanism for penalizing local governments and urge them to actively implement environmental policies.
3. For violations by enterprises, local governments can increase the amount of penalties, and it can also place restrictions on noncompliant enterprises in terms of environmental credit evaluation, loan financing, and access rights to new markets, directly or indirectly raising the losses suffered by enterprises and forcing them to improve their environmental behavior.
4. Local governments should assume the main responsibility for environmental governance, use a combination of incentive-based and constraint policies, and strengthen the supervision and guidance of enterprises. On the one hand, by increasing tax relief, enterprises are incentivized to carry out technological upgrading and equipment renovation to achieve cleaner production. On the other hand, an increase in the environmental protection tax rate will guide enterprises to control their pollution and lower their end-of-pipe emissions.
5. The central government should actively normalize inspections and leverage the advantages of a pressure-based accountability mechanism. At the same time, it is necessary to pay more attention to the role of media supervision, combining media supervision with central environmental supervision and forming strong constraints on the violations of enterprises and local governments to avoid the occurrence of environmental regulatory capture and promote effective pollution control.

## Data availability statement

The original contributions presented in the study are included in the article/supplementary material, further inquiries can be directed to the corresponding author.

## Author contributions

ZH: Methodology, Writing—original draft. YW: Writing—review and editing. HZ: Funding acquisition, Writing—original draft. WL: Data curation, Writing—original draft. TT: Validation, Writing—review and editing.
